# Inclusive Brain: From Neuronal Doctrine to the Active Milieu

**DOI:** 10.1093/function/zqab069

**Published:** 2022-01-10

**Authors:** Alexey Semyanov, Alexei Verkhratsky

**Affiliations:** Shemyakin-Ovchinnikov Institute of Bioorganic Chemistry, Russian Academy of Sciences, Moscow 117997, Russia; Faculty of Biology, Moscow State University, Moscow 119234, Russia; Sechenov First Moscow State Medical University, Moscow 119435, Russia; Sechenov First Moscow State Medical University, Moscow 119435, Russia; Faculty of Biology, Medicine and Health, The University of Manchester, Manchester M13 9PT, UK; Achucarro Center for Neuroscience, IKERBASQUE, Basque Foundation for Science, 48011 Bilbao, Spain & Department of Neurosciences, University of the Basque Country UPV/EHU and CIBERNED, Leioa, Spain

*“We hold these truths to be self-evident, that all cells in the brain are created equal and work in concert thus producing our emotions, and thoughts, and behaviors.”*

Paraphrasing Declaration of Independence of the thirteen United States of America, 1776.

The human brain (as well as brains of other mammals) is a complex multiscale hierarchical structure that spans from individual molecules to organelles, from organelles to cells, which are organised into nuclei and layers, which further form gyri and sulci, lobes, parts, and hemispheres thus making the whole organ covered by meninges and floating in the cerebrospinal fluid within the skull. The nervous tissue is made from the neural cells (neurons, astroglia and oligodendroglia, which are all scions of neuroepithelial cells, and microglia that originates from mesodermal invasion) and non-neural cells that include endotheliocytes, smooth muscle cells and pericytes. All these cells are surrounded by the extracellular space containing extracellular matrix. There are roughly 100 billion neurons in the human brain, ∼ 85 billion of glia, and maybe 5–10 billion endotheliocytes. However, cellular physiology of the brain tends to see every cell population in isolation, with neurons being regarded as the main elements while other cells being subservient to neuronal demands. This is the neuronal doctrine generally associated with Santiago Ramon y Cajal, who, in reality, hold other brain cells in high esteem and never declared neuronal superiority^1^. The preponderance of neuronal doctrine stimulated a retaliatory response from gliobiologists, some of whom proclaimed neuroglia as the primary seat of intelligence^2^.

Evolution casts cellular functions to achieve adaptability. The brain is an example of division of tasks and specialisation between cellular elements that, by operating in concert, create unique tissue tailored for controlling the body, processing sensory inputs, memorising, learning, and producing decisions translating into behaviours. The sensory inputs and motor outputs of the brain are carried by axons joined into nerves, whereas a complex network of different types of brain cells stores and processes information. The brain functions are based on dynamic interposition and interaction of neurons, astroglia, oligodendroglia, microglia, blood vessels, and dynamic extracellular space, filled with interstitial fluids and extracellular matrix. All cellular and non-cellular components of the brain jointly create the active milieu of the brain^3^ (Figure 1). Within the active milieu, signals emanate from and are directed to all components, which respond to these signals and generate feedback.

**Figure 1.**
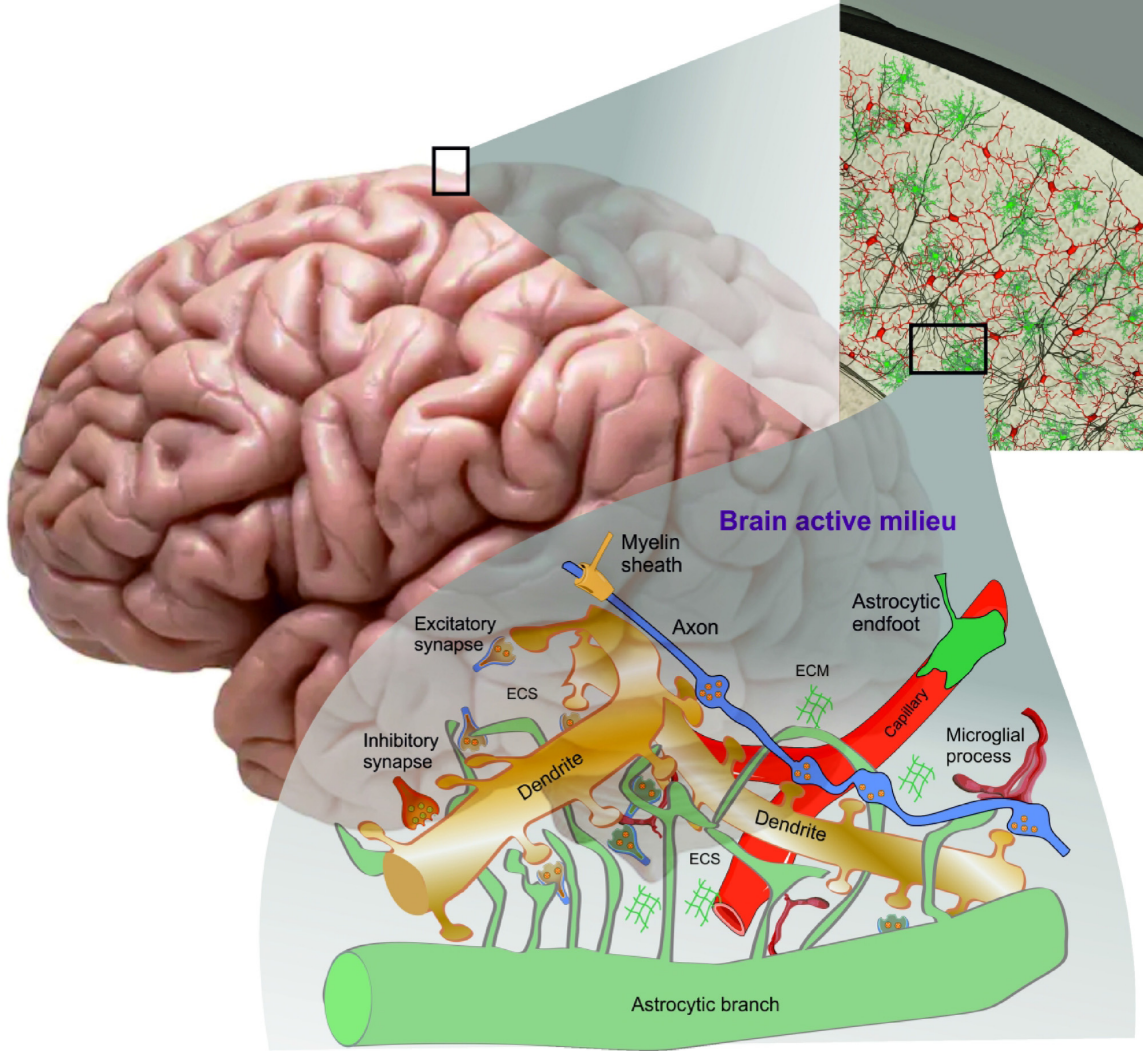
The brain active milieu. The brain as an organ is organised from several interconnected structures: neocortex, hippocampus, thalamus, cerebellum and so on. Each structure is built of multiple cell types (including neurons, neuroglia and non-neural cells) and non-cellular elements (extracellular space with extracellular matrix). All these elements are interlocated in space and interconnected. Specialised connections are formed between cells of the same type (homocellular contacts such as synapses between neurons or gap-junctions between astrocytes) and between cells of different types (heterocellular contacts, for example, neurogliovascular unit or myelinated axon). Astrocytic leaflets fill the space between synapses and respond to the activity of an individual synapse or integrate inputs from several neighbouring synapses. Astrocytic responses and feedback to neuronal structures are influenced by the size and geometry of extracellular space. Extracellular matrix affects neuronal excitability and de novo synaptogenesis, which is also controlled by astrocytes. Synergistic activity of astrocytes and microglia underlie synaptic pruning, which shapes synaptic connectivity in neuronal ensembles. Jointly all these elements form the brain active milieu, which spans from the subcellular level to the level of local microcircuits. Inputs to the active milieu arrive from the neurons connecting the brain to the sensory organs, from vasculature with blood flow and vasomotion, from astrocytes sensing internal brain environment. These inputs inform the brain about the world around and, after being processed by the nervous tissue, instigate adaptive behaviours. Incoming signals evoke plastic remodelling of the brain active milieu that underlies learning and memory. The aggregate of these changes, which affect all elements of the active milieu, can be defined as an engram. Drawn, based, in part, on^3^ with permission.

Interactions in the nervous tissue are multidirectional and mediated through various, often crisscrossing pathways. Central chemo- and baroreception involve, for example, both neurons and astrocytes, which perceive intracranial pressure and brain oxygenation^4–6^ to regulate both local and systemic circulation. Hormones released by endocrine glands, biologically active substances synthesised by microbiota, metabolites produced in the liver as well as immune signalling molecules all arrive in the brain with the blood stream and affect brain functions after passing through brain–blood barrier and interacting with various elements of active milieu^7,8^. At the systemic level the brain participates in a diversity of interactive axes such as brain – intestines – microbiota; brain-heart – blood vessels; brain – immune system and so forth. Within these axes, the brain relies on the inputs from the peripheral nervous system as well as internal environment sensors.

When signals generated by the external world and internal environment arrive in the brain, they get processed by cellular ensembles, which integrate the activity of sub-cellular structures represented by dendrites receiving synaptic inputs, astrocytic and microglial processes, or endothelial-pericytes contacts. Several concepts describing bidirectional interaction of cellular elements have been proposed, including tri- or multipartite synapse, neurogliovascular unit, extrasynaptic signalling, volume transmission, glymphatic flow etc. The inclusive concept of active milieu^3^ integrates all these concepts and postulates that the elementary morpho-functional unit of the nervous tissue is a complex of compartments provided by its cellular constituents.

The operation of the brain active milieu is not a simple summation of elements; their complex interactions generate new quality defined by the plasticity of interelement functional links. For example, each brain state (awake, sleep, and pathological) or form of brain activity (learning, exploring, deciding) requires interactions of all elements of the active milieu. We define several principles of interaction in the active milieu. First, the elements (Figure 1) interact through multiple routes. Second, every single element connects to multiple elements. Third, the same functional outcome can be achieved through different patterns of interactions. These principles predict that multiple links between active milieu elements form a functional interactome of the brain where the elements represent functional nodes. Shaping and modifying the strength of the links is determined by signals arriving in the brain from sensory organs and sensors of the internal environment to form a memory trace, or the engram. Thus, the active milieu extends the basis of engram theory, which was based purely on neuronal plasticity and rewiring of neuronal networks. Outcomes of plastic changes of active milieu elements and their interaction routes are more complex than those in a network made solely from neurons. For example, the morphological plasticity of an astrocyte can simultaneously affect local multipartite synapses, neurogliovascular units and extracellular space. Plastic remodelling of an individual neuron or its parts changes its interactions with other neurons, astrocytes and extrasynaptic signalling routes thus dynamically remodelling the active milieu.

Active milieus are many, and they are region specific reflecting different properties of cells in different parts of the brain. For example, in the cerebellum, radial Bergmann astrocytes create distinct perisynaptic covers organised into an array of appendages emanating from the main processes^9^; this structure is very much different from leaflets covering synapses in the neocortex. The functional expression of receptors is another variable that defines functional interactions between elements. Finally, pathology acts by reshaping the active milieu through, for example, cellular reactivity or by the invasion of blood-born factors^10^, which alter morpho-functional interactions and thus affect the operation of the nervous tissue.

Description of brain function based on organisation and plasticity of the neuronal circuit provided useful conceptual framework for testable hypothesises. These hypotheses have been routinely examined in reduced systems such as neuronal cultures or brain slices. The development of techniques for *in vivo* experiments that simultaneously interrogate different cell types requires a different conceptual framework, which considers multiple interactions among brain cell types within the multicellular physiological environment. The concept of the brain active milieu may redefine the experimental design to functional links of all cellular and non-cellular elements of the nervous tissue by probing multiple interaction routes in which each target element is involved.

## Funding

This work was supported by the Russian Foundation for Basic Research (RFBR), Grant No. 21-54-53018.
